# Hooked epinephrine auto-injector devices in children: four case reports with three different proposed mechanisms

**DOI:** 10.1186/s13223-020-00418-0

**Published:** 2020-03-14

**Authors:** Ran D. Goldman, Katharine C. Long, Julie C. Brown

**Affiliations:** 1 The Pediatric Research in Emergency Therapeutics (PRETx) Program, Division of Pediatric Emergency Medicine, Department of Pediatrics, University of British Columbia, BC Children’s Hospital Research Institute, 4480 Oak St, Vancouver, BC Canada; 2 Mary Bridge Children’s Hospital, Tacoma, WA USA; 3 Division of Pediatric Emergency Medicine, Department of Pediatrics, Seattle Children’s Hospital, University of Washington, Seattle, WA USA

**Keywords:** Anaphylaxis, Epinephrine auto-injectors, Auto-injector complication, Hooked needles, Children

## Abstract

**Background:**

The prevalence of epinephrine auto-injectors (EAI) use is on the rise. Our objective was to describes children with hooked EAI needles that were embedded in soft tissues.

**Case presentation:**

Results: Two children self-injected in their shins. The embedded EAIs required removal in the Emergency Department. Both needles were hooked and splayed at the tip. A boy in anaphylaxis kicked his leg during EAI injection and the hooked needle embedded under his skin and was difficult to dislodge. The exposed needle was curved. A girl had an EAI administered for anaphylaxis, which was also difficult to dislodge. On removal, the distal needle tip was hooked approximately 160 degrees. Images of the device revealed that the needle fired off-center from the device and the device components were cracked. We propose three different explanations for these hooked EAI needles. The first is that the needle could hit bone during injection and curve rather than penetrates further. Secondly, the needle could bend when the patient moves during injection. Thirdly, if a needle fires sufficiently off-center to hit the cartridge carrier, this could hook the needle prior to injection.

**Conclusions:**

Awareness of the reasons for needle hooking, damage observed, and challenges and successful approaches to their removal, can better prepare the provider for these uncommon events. Teaching parents, children and educators about safe EAI storage and appropriate restraint during use may prevent some of these accidental injuries. Reporting device failures may lead to improvements in device performance and design.

## Highlights

What is already known about this topic?

Prevalence of anaphylaxis is on the rise and with it the use of epinephrine auto-injectors (EAIs). Complications associated with EAIs include lacerations and digit injection.

What does this article add to our knowledge?

We describe an uncommon complication of using EAIs in children—hooked needles that were embedded in soft tissues, and provide potential explanations to this phenomenon.

How does this study impact current management guidelines?

Teaching parents, children and educators about safe EAI storage and use may prevent accidental injuries and increase awareness of the reasons for needle hooking in order to better prepare for these uncommon events.

## Background

During the past 50 years, the prevalence of anaphylaxis has increased and with it the use of epinephrine auto-injectors (EAIs) [1–3]. Rare complications associated with EAIs include thigh lacerations, digit injection and embedded needles [4].

We describe four children with hooked EpiPen needles that were embedded in soft tissues. Three cases came from investigators’ institutions, and the fourth was reported to one of the investigators via social media. The cause of the needle hooking likely differed between cases. All families provided written consent to publish this report.

## Description of cases

### Case 1

**Fig. 1:**
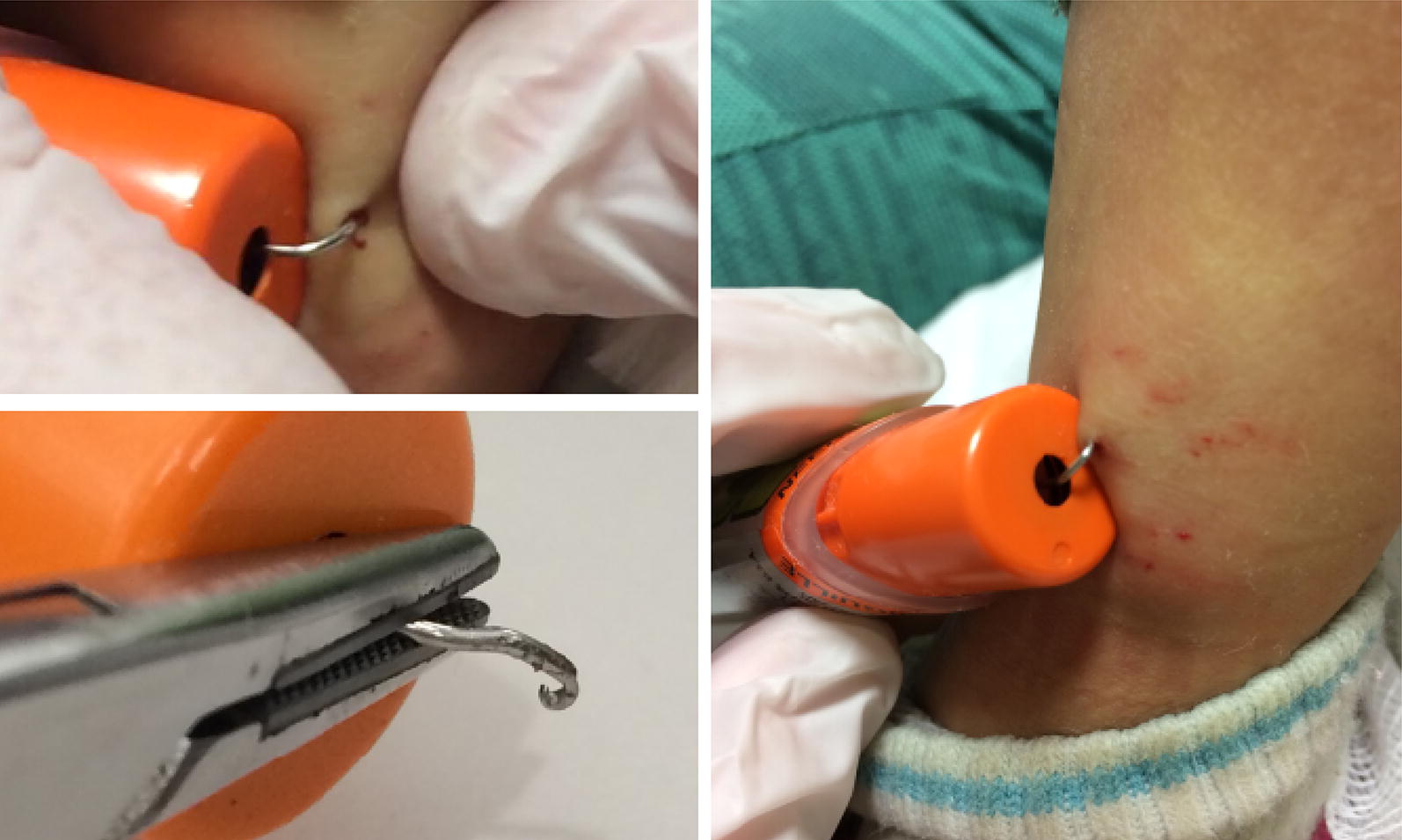
Case 1

A healthy, non-allergic 7-year-old girl found an epinephrine auto-injector in her school playground and decided to test its action by injecting into her left mid-shin. She was unable to remove the device and was brought to the Emergency Department (ED) by Emergency Medical Services (EMS), with the EpiPen still attached to her leg (Fig. 1).

Upon arrival, the EpiPen was hanging from the skin. The child was calm and alert, anxious but in no apparent pain. Her heart rate was 130/min, respiratory rate 20/min, Oxygen saturation 100% in room air.

She was given combined analgesia and anxiolysis that included Child Life Specialist guidance, a squeezable sponge in her hand, Virtual Reality goggles with an animated roller coaster app, and injected local 1% lidocaine (3 ml) around the area of the EpiPen needle. The needle was then successfully removed. Following removal, the needle was noted to be hooked 180 degrees with a split tip (Fig. 1). No further treatment was required and the child was discharged shortly thereafter.

### Case 2

**Fig. 2:**
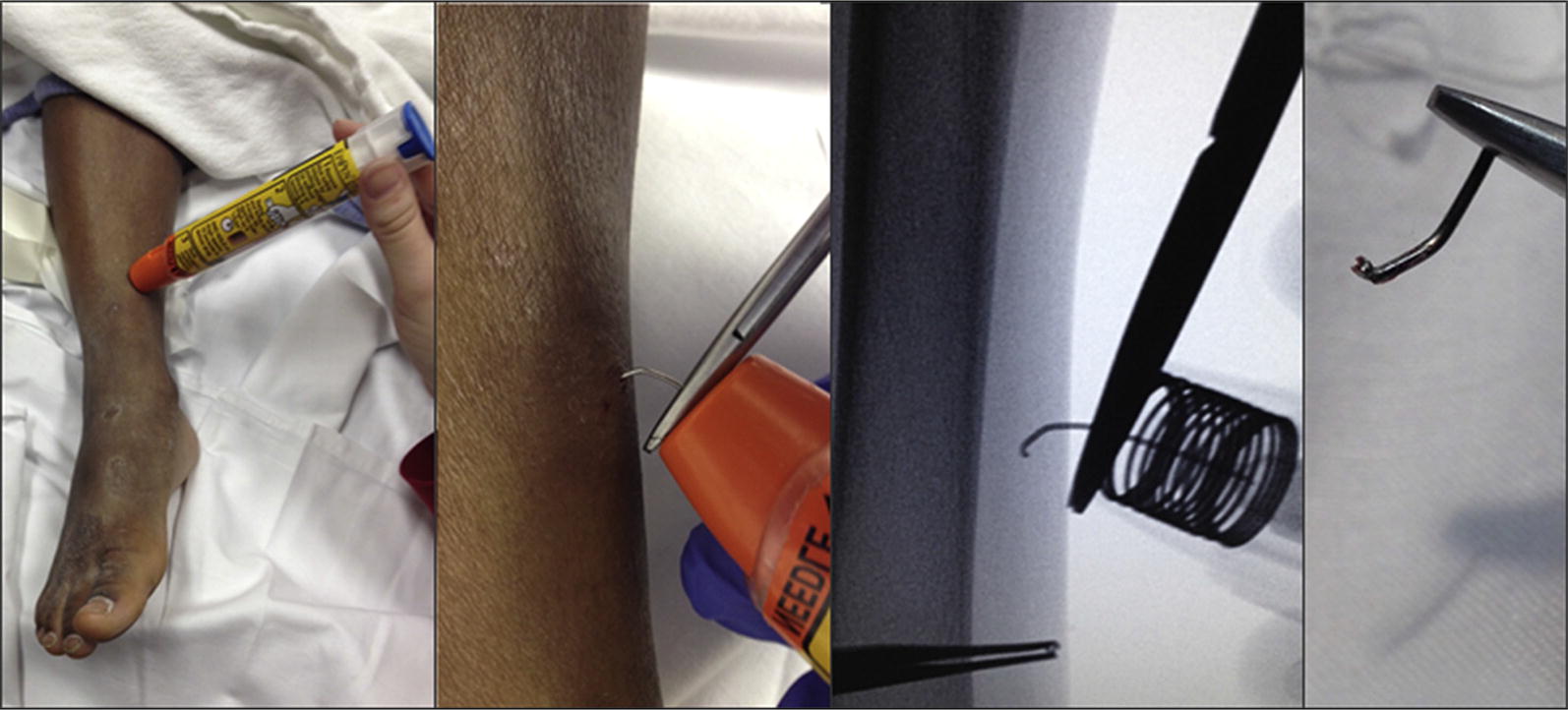
Case 2

A 5-year-old boy found a relative’s EpiPen in his house and accidentally injected himself in the lower shin (Fig. 2). His family and EMS providers were unable to remove the device and he was transported to a pediatric ED. Examination under fluoroscopy revealed that the needle was bent underneath the child’s skin. After 1% lidocaine was injected locally, the needle still could not be easily extracted. The proximal end of the needle was cut free from the device and the distal tip was manipulated up, poked through the skin, and removed. Fluoroscopic images of the needle prior to removal, as well as photographs following removal, revealed that the needle was hooked and the tip was split. No further treatment was required, and the child was discharged. This case has been reported previously [4].

### Case 3

**Fig. 3:**
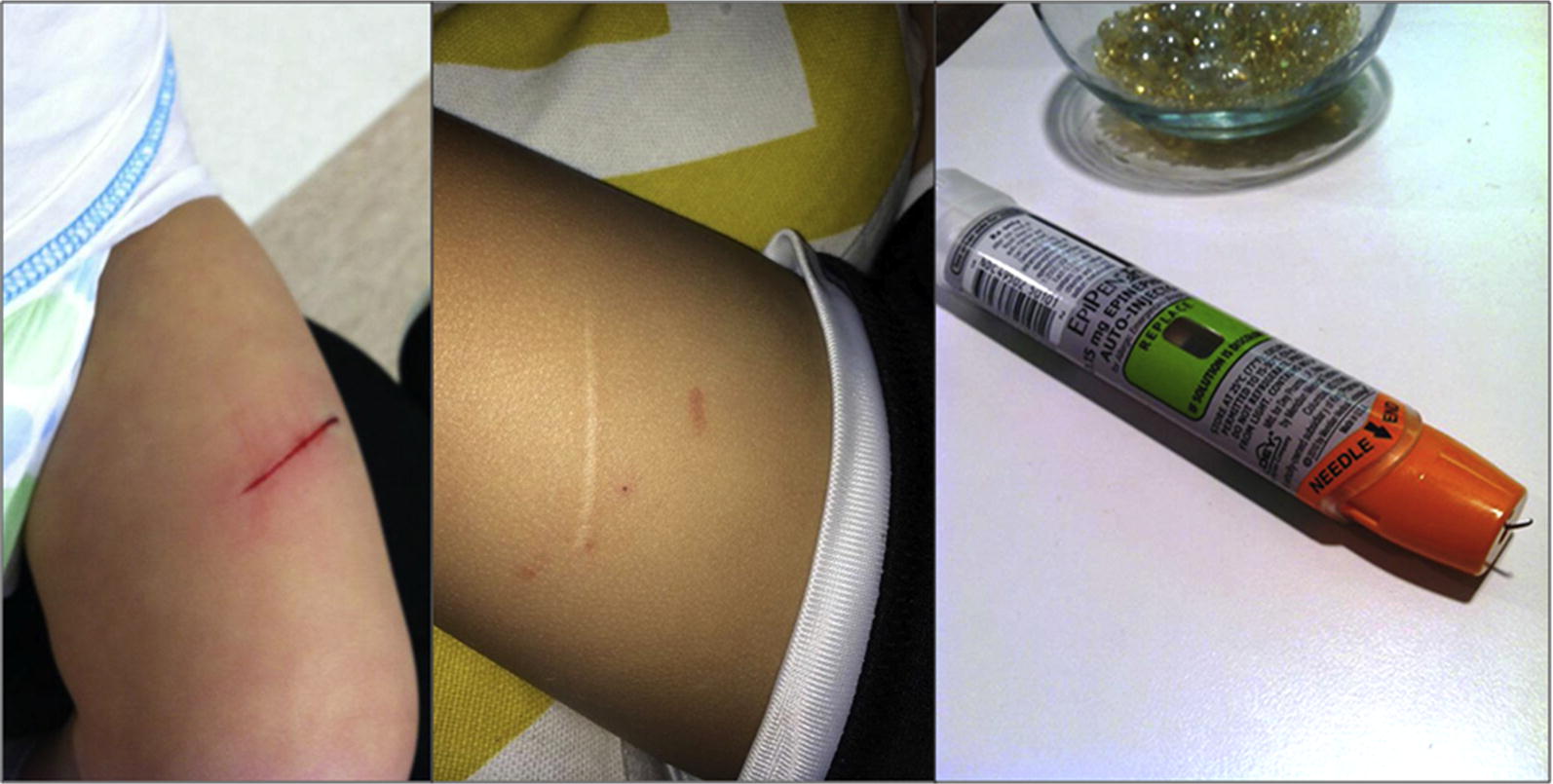
Case 3

A 16-month-old boy developed an allergic reaction while eating at a restaurant. His mother held her son on her left hip and injected an EpiPen Jr with her right hand against his left thigh, using a push-and-hold approach (Fig. 3). He initially did not react to the injection, but after a few seconds, he became more responsive and began to kick his leg, resulting in a 3-cm laceration of his left thigh. His mother described the needle as “stuck like a hook” under his skin and she was initially unable to remove it. She had to insert it further to finally work it free. On removal, the needle was curved and uncovered. This case has been reported previously [4].

### Case 4

**Fig. 4:**
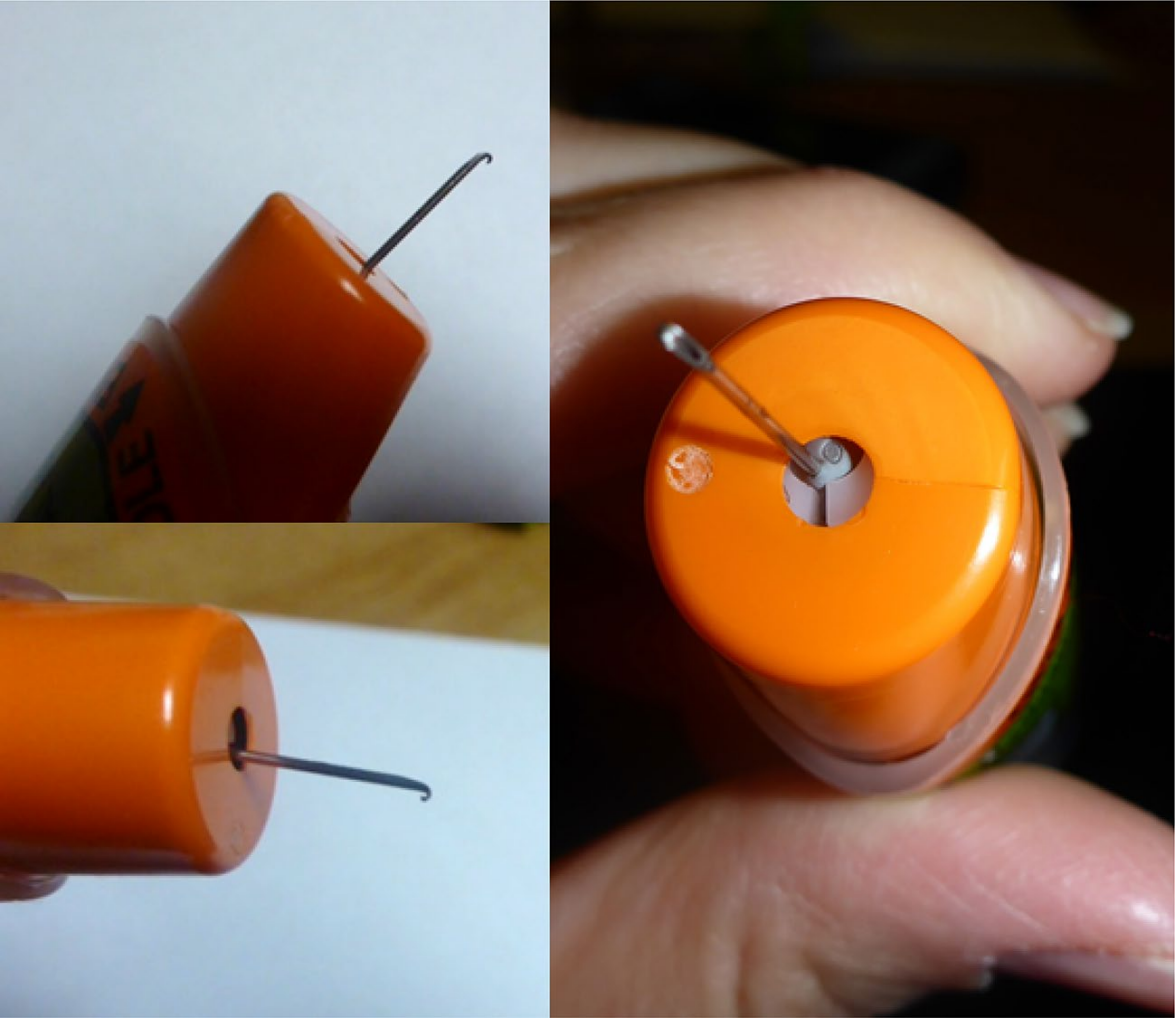
Case 4

A 4-year-old 15 kg girl had symptoms of anaphylaxis. An EpiPen was administered by her mother at home, in her lateral thigh (Fig. 4). The patient was well restrained by her father during injection, and per parental report she did not move at all during the injection. Her mother reported that it felt like it was stuck in the muscle when she tried to pull it out, and she had to ‘pull hard’. It then stuck again in the skin and had to be further dislodged until it finally came free. Images of the removed device revealed that the needle did not fire out of the center of the device, that it pierced the rubber needle cover off-center, the white carrier and orange needle shroud were both cracked, and the needle tip was hooked. The patient sought care for her anaphylaxis, but the injection site did not require any intervention.

## Discussion

The most common injuries reported with EAIs are unintentional injections. The incidence of accidental injection, mostly involving EpiPen devices, which are the most commonly used on the market, injected into the thumb, is estimated at 1 in 50,000 EpiPen units [5], and up to 16% of doctors who read the instructions on the autoinjector used the EpiPen^®^ trainer in a manner that would have self-injected into their thumb [6]. There were over 15,000 unintentional EpiPen injections reported to U.S. Poison Control Centers over 14 years of study [7]. Of 105 unintentional injections from EAIs reported to the Food and Drug Administration Adverse Event Reporting System [7], more than one-third of the individuals injected were health care professionals.

Lacerations and injuries from epinephrine auto-injector needles are less common but pose a risk. Brown et al. reported 25 cases of EpiPen-associated laceration and embedded needle injuries [4] including 20 with thigh lacerations, a nurse with a digit laceration, and four children with stuck needles. The mean age in injured children was 3 years. Operators included parents, educators and a child, but also involved healthcare providers. The authors suggested that the 10-second hold of the EpiPen may have contributed to these injuries and may be excessive, considering evidence that EpiPens deliver epinephrine in less than 3 s [8, 9]. The hold time for EpiPen was subsequently reduced to 3 s in the United States. The hold time varies in other countries between 3 s (England and Australia), several seconds (Canada), 5 s (Sweden) and 10 s (many European, African and Asian countries).

Brown and Tuuri reported an additional case of laceration and provided guidance for providers on how to educate families regarding appropriate child restraint during injection [10]. In the United States, patient information now includes instructions to “hold the child’s leg firmly in place and limit movement prior to and during injection” (accessdata.fda.gov/drugsatfda_docs/label/2017/019430s067lbl.pdf), although these simple instructions may fail to convey the degree of restraint needed to prevent these injuries in a combative child.

While bent needles are often reported in connection with leg laceration injuries, hooked needles are a less frequent complication of EpiPen use. Two of the current cases of hooked EpiPens were reported previously [4], however, the cause of and management of hooked EAI needles has not been discussed elsewhere. We propose three explanations for the hooked EpiPen needles observed in this study. The first is that the needle could hit a hard structure such as bone during injection and curve rather than penetrate further. This may explain the first 2 cases described here, where the EpiPen was injected in an area with a short skin-to-bone distance. This type of hooking might similarly happen if the needle were to hit a very stiff seam of clothing, although we are unaware of any reports of this occurring. It is unlikely that a plain film would identify the location of impact, so x-rays of the bone are unlikely to offer proof of this proposed explanation. Instructions indicate that users should avoid injecting at closing seams [11]. Secondly, the needle could bend if the patient moves during injection. In most cases, patient movement results in needles bending in one straight line or with a simple curve, rather than a true hook [4]. However, we describe one case where the tip of the curved needle appeared and behaved “like a hook”. Thirdly, our experience with testing many EpiPen devices suggests that EpiPens needles frequently don’t eject from the device perfectly straight. If they are sufficiently off-center to hit the cartridge carrier, this could hook the needle prior to injection.

In the fourth case we present, it is most likely that the needle became hooked prior to injection. The needle pierced the side rather than the center of the rubber needle cover, and then appears to have made contact with, and cracked, the white carrier that houses the cartridge and stopper. It then also appears to have cracked the orange shroud that typically covers the needle upon removal from the body. These two contacts appear to have hooked the needle, which likely entered the patient that way. The hooked needle was then difficult to remove.

The ergonomics of the EpiPen has attracted some concerns in the past [12]. Upside-down use of EpiPen devices resulting in thumb injections as well as failed drug administration has been frequently reported. Suboptimal ergonomic design was cited as a reason for about half of cases of more than 100 unintentional injections, as people were trying to self-inject or inject to another person having an allergic reaction [10].

Some proposed changes to administration of EpiPen may improve its safeuse [4, 13].

It is hard to determine what role needle bending plays in the creation of laceration injuries in children in other cases, but in two of our cases no laceration was noticed and the needle insertion site healed well. Bent needles are not covered by the plastic casing, which poses a potential injury to children and EAI providers. While minimizing needle injection time may have prevented some lacerations described previously [4], it is hard to predict if this would have been preventing the injury in the cases we present here.

Hooked needle were only seen with EpiPen devices in this study. This may reflect device prevalence in the community. Two other EAIs are available in the United States: Auvi-Q (kaléo, Richmond, VA) and a generic for Adrenaclick (Amneal Pharmaceuticals, Bridgewater, NJ.). In these devices, the syringe does not push a rubber stopper up against a cartridge carrier during firing. The mechanisms of firing are sufficiently different that they may not have the same potential for off-center firing of the needle compared with the EpiPen device.

## Conclusion

Hooked needles are a rare potential hazard from using the EpiPen, mostly associated with incorrect use of the device. Teaching parents, children and educators about safe EAI storage and use may prevent accidental injuries. Awareness of the reasons for needle hooking, the needle damage observed, and approach to their removal, can better prepare the provider for these uncommon events. One case was associated with a suspected device malfunction. Reporting EAI problems to the Food and Drug Administration via their provider and consumer reporting program may lead to improvements in device performance and design.

## Data Availability

All data is available with the authors.

## Abbreviations

EAIs: Epinephrine auto-injectors
ED: Emergency Department
